# Validation of γ-radiation and ultraviolet as a new inactivators for foot and mouth disease virus in comparison with the traditional methods

**DOI:** 10.14202/vetworld.2015.1088-1098

**Published:** 2015-09-19

**Authors:** Safy El din Mahdy, Amr Ismail Hassanin, Wael Mossad Gamal El-Din, Ehab El-Sayed Ibrahim, Hiam Mohamed Fakhry

**Affiliations:** Department of Foot and Mouth Disease, Veterinary Serum and Vaccine Research Institute, Abbasia, P. O. Box. 131, Cairo, Egypt

**Keywords:** A/Iran05 and SAT-2/2012, binary, combination (BEI+FA), enzyme linked immunosorbent assay (ELISA), foot and mouth disease virus, gamma radiation, heat, inactivation, ISA201, O/pan Asia, ultraviolet light, vaccine formulation, serum neutralization test

## Abstract

**Aim:**

The present work deals with different methods for foot and mouth disease virus (FMDV) inactivation for serotypes O/pan Asia, A/Iran05, and SAT-2/2012 by heat, gamma radiation, and ultraviolet (UV) in comparison with the traditional methods and their effects on the antigenicity of viruses for production of inactivated vaccines.

**Materials and Methods:**

FMDV types O/pan Asia, A/Iran05, and SAT-2/2012 were propagated in baby hamster kidney 21 (BHK21) and titrated then divided into five parts; the first part inactivated with heat, the second part inactivated with gamma radiation, the third part inactivated with UV light, the fourth part inactivated with binary ethylamine, and the last part inactivated with combination of binary ethylamine and formaldehyde (BEI+FA). Evaluate the method of inactivation via inoculation in BHK21, inoculation in suckling baby mice and complement fixation test then formulate vaccine using different methods of inactivation then applying the quality control tests to evaluate each formulated vaccine.

**Results:**

The effect of heat, gamma radiation, and UV on the ability of replication of FMDV “O/pan Asia, A/Iran05, and SAT-2/2012” was determined through BHK cell line passage. Each of the 9 virus aliquots titer 10^8^ TCID_50_ (3 for each strain) were exposed to 37, 57, and 77°C for 15, 30, and 45 min. Similarly, another 15 aliquots (5 for each strain) contain 1 mm depth of the exposed samples in petri-dish was exposed to UV light (252.7 nm wavelength: One foot distance) for 15, 30, 45, 60, and 65 min. Different doses of gamma radiation (10, 20, 25, 30, 35, 40, 45, 50, 55, and 60 KGy) were applied in a dose rate 0.551 Gy/s for each strain and repeated 6 times for each dose. FMDV (O/pan Asia, A/Iran05, and SAT-2/2012) were inactivated when exposed to heat ≥57°C for 15 min. The UV inactivation of FMDV (O/pan Asia and SAT-2) was obtained within 60 min and 65 min for type A/Iran05. The ideal dose for inactivation of FMDV (O/pan Asia, A/Iran05, and SAT-2/2012) with gamma radiation were 55-60 and 45 kGy, respectively. Inactivation of FMDV with binary was 20, 24 and 16 hr for O/pan Asia, A/Iran05, and SAT-2/2012, respectively while inactivation by (BEI+FA) was determined after 18, 19 and 11 hr for O/pan-Asia, A/Iran 05, and SAT-2/2012, respectively. The antigenicity of control virus before inactivation was 1/32, it was not changed after inactivation in case of gamma radiation and (BEI+FA) and slightly decrease to 1/16 in case of binary and declined to 1/2, 1/4 in case of heat and UV inactivation, respectively. The immune response induced by inactivated FMD vaccines by gamma radiation and (BEI+FA) lasted to 9 months post-vaccination, while the binary only still up to 8 months post-vaccination but heat and UV-inactivated vaccines were not effective.

**Conclusion:**

Gamma radiation could be considered a good new inactivator inducing the same results of inactivated vaccine by binary with formaldehyde (BEI+FA).

## Introduction

Foot and mouth disease (FMD) is a contagious viral disease affecting cloven-hoofed animal that causes highly significant losses. Therefore; using safer and high potent vaccines are required [1]. Decrease in milk production, weight gain, reproductive inefficiencies, and death in young ruminants is the main economic losses caused by FMD [2,3]. The virus inactivation process is the most safety and critical steps in the production of FMD vaccines. For FMD vaccines, in particular, guaranteed safety is essential because any occurrence of the disease will have great economic consequences [4].

FMD vaccines used around the world are inactivated vaccines for prophylactic or emergency use, generally manufactured by the same basic methodology outlined in the OIE manual [5].

Inactivation can be performed using chemical or physical methods or a combination of the two. A wide range of well-established and novel inactivation methods have been described to successfully inactivate viruses for vaccine purposes. Examples are formaldehyde [6,7], binary ethyleneimine (BEI) derivatives [8], sodium chloride or phosphate [9], psoralens [10], hydrogen peroxide [11], heat [12-14], ultraviolet (UV) irradiation [13-16], and gamma radiation [17-20].

Thermal and UV irradiation’s inactivation are depending on a function of dose, where in UV depending on intensity and time. The UV dose is defined and measured as incident energy (not absorbed energy) [21]. The decimal inactivation dose is defined as the amount of UV irradiation required to minimize the number colony of microorganisms by a factor of 10% or 90% [16].

The old history of FMD vaccine was in the form of virus inactivated with formalin. However, the main disadvantage of formalin is altering the structure of the virion [22]. So, production laboratories changed to aziridine group inactivators like acetyl ethylenamine as inactivator [23,24]. Inactivation with BEI is used in particular, because this method developed by [25,26] circumvents the direct handling of the very toxic other aziridine.

There are limited studies on UV inactivation of FMD virus (FMDV) using irradiation.

The present study was designed to investigate the effects of heat, γ-radiation, and UV on foot and mouth virus strains (O/pan Asia, A/Iran05, and SAT-2/2012) inactivation, in addition to evaluate the immune response induced by prepared vaccines using such inactivators in comparison with traditional prepared BEI and/or formaldehyde inactivated vaccine.

## Materials and Methods

### Ethical approval

The experiment was as per the protocol of Institutional Animal Ethics Committee, the authors had taken permission of animal owners of the private farm.

### Tissue cultures

#### Baby hamster kidney cell line (BHK 21)

BHK21 was obtained from the World Reference Lab. Pirbright Surrey, U.K. These cells were used for virus propagation, virus titration as well as confirmation of complete inactivation process.

#### Virus multiplication

FMDV types O/pan Asia, A/Iran05, and SAT-2/2012 were propagated in BHK21 monolayer cell cultures then incubated at 37°C for about 18-20 hrs till the observation the morphological changes in cell (cytopathic effect [CPE]) according to Declarq *et al*. [2]; Longjam *et al*. [27]. The virus was centrifuged at 3000 rpm for 15 min, and the supernatants were separated and dispensed in 2 ml glass vials and stored at −70°C till used [28,29].

#### Titration of virus by tissue culture

Serial ten folds dilutions of FMDV were prepared in tissue culture plates using Hank’s solution, 25 µl/well, from each dilution a set of 4 wells were inoculated on BHK cells and control non-infected cells were inoculated with 25 µl of Hank’s solution then the plate was incubated at 37°C for 2 days and observed for the cytopathic changes (CPE) and compared with the control non-infected cells. Finally, the titer was expressed as log_10_ TCID_50_ as described by Reed and Muench [30].

#### Chemicals used for inactivation

Bromoethylamine hydrobromide (BEA)

It was obtained from Aldrich Chemical Company Limited Gillinham, Dorest, U.K.

Sodium hydroxide (Analar) NaOH (molecular weight = 40)

It was obtained from PRATAP Chemical Industries Pvt. Ltd (India) was used in concentration of 0.2 normality for dissolving (BEA) in cyclization process according to Bahneman [25].

Formaldehyde 40%

It was obtained from BDH Chemicals Likited Poole, U.K. It’s molecular weight was 30.03, It was used in a concentration 0.04% in inactivation process according to Farid *et al*. [31].

Sodium thiosulfate (Na_2_S_2_O_3_.5H_2_O)

Of molecular weight (248.18), 20% solution in double distilled water was prepared and sterilized by autoclaving. The chemical obtained from Meck Company, Germany. It was used in a final concentration of 2% to neutralize the excess of BEA after the inactivation process as described byGirardn *et al*. [32].

Sodium bisulfite (Na_2_S_2_O_5_)

Its molecular weight was (190.10) obtained from Merck Company, Germany. It was prepared as 20% solution, sterilized by autoclaving, and used in a final concentration of 2% to neutralize the excess of formaldehyde after the inactivation process according toFarid *et al*. [31].

#### Inactivation of FMDV (O/pan Asia, A/Iran05, and SAT-2/2012)

Inactivation by heat

The FMDV strains “O/pan Asia, A/Iran05, and SAT-2/2012” virus (10^8^ TCID_50_/ml) were subjected to various temperatures for varying times. The virus suspension degrees of temperatures distributed in nine tubes of 3 ml capacity for each strain, each containing 2 ml of virus suspension. The tubes were exposed to 37, 57, and 77°C, individually. All samples of each tube were collected after 15, 30, and 45 min intervals.

Inactivation by gamma irradiation

Doses of gamma ray: 10, 20, 25, 30, 35, 40, 45, 50, 55, and 60 kGy were used for irradiation of virus samples with dose rate 0.551 Gy/s. The irradiation was done at 0-4°C by some artificial ices.

Inactivation of FMDV by UV light

The virus suspension was dispersed in sterilized petri-dishes and exposed to UV light source (252.7 nm). A virus sample was collected at 15, 30, 45, 60, and 65 min intervals.

Inactivation by binary (BEI)

Using BEI only, BEI was prepared as 0.1 M in 0.2 N NaOH, with final concentration of 0.001 M (1 mM of BEI) at 37°C for 24 hrs at PH 8.0 according to Bahneman [26]. Sodium thiosulfate 20% was added to treat virus after inactivation in a final concentration of 2% (during 24 hrs) to neutralize the effect of BEI.

Inactivation by binary with formaldehyde (BEI+FA)

The use combination of BEI 1 mM and 0.04% FA (BEI+FA) was carried out according to Barteling *et al*. [4]. Sodium thiosulfate 20% was added to the virus after the inactivation in a final concentration of 2% (up to 24 h), also sodium bisulfite 20% was added after inactivation to neutralize the rest of formaldehyde.

#### Safety test

In cell culture

Infectivity of inactivated viruses with different inactivator was studied by cell culture methods. All samples were inoculated on BHK21 cell lines for three passages and the virus titer was determined.

In baby-suckling mice

The inactivated viruses were inoculated into suckling mice to confirm the complete inactivation of the FMDV. 5 Albino baby-suckling mice 2-4 days for each inactivated virus sample were injected with 100 µl intraperitonially. Reading was recorded until the 5^th^ day post-inoculation [33].

Mice still alive mean complete virus inactivation but death indicates incomplete inactivation.

Complement fixation test (CFT)

CFT was done according to Health Protection Agency [34]. With tested antigen (antigen used in vaccine preparation).

It was necessary to titrate the complement to know the minimum hemolytic dose which will give 100% hemolysis. An eppendorf tube containing 0.2 ml of complement was kept at −70°C and dissolved in 1.8 ml veronal buffer to obtain dilution became 10%. Further dilutions were made to give a dilutions corresponding to 0.5%, 1%, 1.5%, and up to 6%. From each dilution, 25 μl were added to each well of CFT microplate containing 25 μl of the undiluted antigen.

25 μl of veronal buffer and 50 μl of the hemolytic system (HS) were added. One control well containing 75 μl veronal buffer and ml (HS) was incorporated. The plate was shaken well and incubated at 37°C for 15 min. The plate was read visually, and the lowest percentage of complement giving 100% hemolysis of sheep red cell was considered as the accurate does of complement for that antigen which used in the test proper.

### The test proper

25 μl of the undiluted antigen and two-fold dilutions of antigen sample in veronal buffer were added to each well and then 25 μl of the reference guinea pig hyperimmune serum was added followed by 25 ml of complement. Two wells were used as a control, the first one received 25 μl of antigen, 25 μl of complement and 25 μl of veronal buffer. The 2^nd^ one received 25 μl of antigen and 50 μl of veronal buffer. The plate was incubated at 37°C for 30 min, then 50 μl of the HS was added for each well followed by another incubation at 36°C for a min. A positive reaction is shown when there is no hemolysis in the wells.

#### Preparation of inactivated FMD vaccines

The vaccine formulation was carried out according to the method described by OIE [33] where the oil phase consisted of Montanide ISA 201 mixed with the same weight of inactivated viruses (weight/weight) and mixed thoroughly. The vaccine was prepared on the base that each dose (3 ml) of vaccine contains not less 10^8^ TCID_50_/dose and 2.2 µg antigen pay loaded of each virus type.

#### Potency of the inactivated vaccines in calves

Thirty calves free from FMD were divided into six groups (five calves/group). Five groups were inoculated I/M by the inactivated prepared vaccines, and one group was kept as a control without vaccination. Serum samples were obtained from all groups of vaccinated calves every month for 9 months.

Serum samples were examined against FMDV strain (O/pan Asia, A/Iran05 and SAT-2/2012) using serum neutralization test (SNT) and enzyme-linked immunosorbent assay (ELISA).

#### SNT

The test was performed by the microtechnique as described by Ferreira [35] to estimate antibodies against FMD in sera. In flat bottom tissue culture microtiter plates, two-fold serially diluted sera in Modified Eagle’s medium were used. From each dilution, 50 µl per well was distributed into 4 wells, and then 50 µl of the virus was added (100 TCID_50_/ml). Neutralization was allowed to occur at 37°C for 1 h. BHK cells were added (150 µl per well). The test included normal cultures; serum and virus control and incubated at 37°C for 48 hrs with the daily microscopic examination. The SN titer was expressed as the log_10_ of the final serum dilution which protected 50% of wells as calculated by Reed and Muench [30]. For staining of the SNT microplates used in the test, the following procedures were applied.

The media were discarded, and the cell cultures were stained by 1% crystal violet stain for 10 min after which excess stain was discarded, the plates were washed with distilled water for at least 5 times and left for 30 min to dry in the incubator.

#### Indirect ELISA

It was done according to Hamblin *et al*. [36] to determine FMD antibodies in the sera of vaccinated sheep as follows:

Preparation of ELISA antigen

FMDV type O/pan Asia, A/Iran05, and SAT-2/2012 were propagated in BHK_21_ clone 13-cell culture. When 80% CPE was observed, each of infected cell cultures was frozen and thawed three times to release FMDV extracellular.

The virus suspension was centrifuged at 2000 rpm in a cooling centrifuge for 10 min, the supernatant was collected and concentrated by polyethylene glycol - 6000.

Titeration of the conjugate

ELISA microtiter plate was coated with 100 µl of coating buffer for each well. The plate was incubated overnight at 4°C and then was washed with washing buffer at least 5 times. The plate was blocked with (phosphate buffered saline [PBS]) with 2% bovine albumin and incubated overnight at 4°C.

(Serial dilutions of the conjugate (Rabbit anti-bovine immunoglobulin G, horseradish peroxidase labeled), from 1/1000 up to 1/22000 were made in the diluting buffer (PBS with bovine albumin).

An amount of 50 µl of each dilution was placed in a vertical column of the wells in a 96 flat-bottomed microplate. By means of a micropipette, 50 µl of the working solution of orthophenylene diamine (OPD) substrate, freshly prepared, were added to all wells. The plate was covered and placed on a shaker at slow speed (1000 rotate/min) at a dark place. The reaction was stopped by adding 25 µl. Of sulphoric acid (1.25 mol) to each well. The plate was read on ELISA reader at a wavelength of 492 nm. The end point of the dilution should be above 1.0 optic density (OD). The titer of the conjugate was 1/20,000.

Antigen titeration

The antigen is titrated to determine the optimum concentration of the antigen to be used in the assay. This was determined by the checkerboard method. Titration of the diluted antigen ran against positive and negative sera.

Serial dilutions of the FMD antigen (virus strain) as 1/10, 1/20, up to 1/200, were made using the coating buffer solution. 100 µl from each dilution were transferred to two successive horizontal column plates. The plate was covered and incubated overnight at 4°C. The sensitized plate was forcefully decanted and washed five times with the washing buffer (PBS tween) and dried by tapping it upside down on a filter paper. In clean tubes, serial two-fold dilution of the positive (+ve) and negative control sera were made using PBS with 1% bovine albumin as a diluting buffer, starting with 1/4 up to 1/256. The first vertical two rows were left as blank. 100 µl. Per well of strong +ve serum dilutions were transferred horizontally to rows numbers (3,4), (5,6), (7,8), (9,10), and (11,12) that corresponding to antigen dilutions 1/10, 1/20, 1/40 up to 1/320, respectively. The last horizontal row (H) received 100 µl per well from H3 up to H12 as negative control serum. The plate was incubated at 37°C for 1 h after which the contents were decanted, and the plate was washed 3 times and dried as before. 100 µl of the diluted conjugate solution (previously titrated) were added to each well. The plate was reincubated at 37°C for 1 hr then decanted, washed, and dried as before. After that, 100 µl of the freshly prepared substrate (OPD) were added to each well. The plate was covered and shaked on a shaker at slow speed for 15 min at the dark place after which, the reaction was stopped by adding 25 µl. of the stopping solution 1.25 mol (sulphoric acid) to each well. The plate was then read using ELISA reader at wavelength 492 nm.

The highest dilution of virus antigen which gave 1.9 OD with the positive control serum was used in the test proper.

The test proper

Sera collected from the experimentally inoculated sheep were tested for FMD antibodies using the ELISA technique.

Coating: ELISA plates were coated with the FMD antigen by adding 100 µl of 1/140 diluted antigen in carbonate-bicarbonate buffer (according to the titer) in the 96 flat-bottomed wells. The plate was then incubated at 4°C overnight, after that the plate contents were decanted, and the plate was washed three times with the washing buffer and dried as described before.

Blocking: The coated plate was blocked by adding 100 µl of blocking buffer (PBS buffer with 3% serum bovine albumin) per well and incubated overnight at 4°C, then the contents were decanted, the plate was washed and dried as before.

Serum dilutions: To each well of the coated plate, 90 µl were added then 10 μl of the tested serum were added to the first well to make a final dilution 1/10. Each serum sample was run in duplicates, including the control positive (strong and weak positive sera) and negative sera, as well as, the blank control. The plate was covered and incubated at 37°C for 1 h.

Addition of the conjugate: The content of the plate was decanted, washed three times using washing buffer, and then 100 µl of the diluted conjugate 1/20000 were added to all the wells. The plate was covered and incubated at 37°C for 1 h.

Addition of the substrate: The content of the plate was decanted and washed 3 times with the washing buffer solution. Then 100 µl of the substrate OPD were added to each well. The plate was covered and incubated in the dark place for 15 min at 37°C. A brownish coloration indicating positive reaction was developed.

Addition of the stopping solutions: The reaction was then stopped by adding 25 µl per well of 1.25 mol of sulphoric acid, and the plate was read using ELISA reader at 492 nm.

Interpretation of the results: Mean of the OD of sample or control


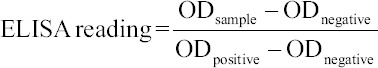

- The result may be 1.0 or more than 1.0 or <1.0.
- Ratio 1.0 or more means positive, <1.0 means negative, cut off of FMD (positive control serum dilution) is 1.9.

## Results

The inactivator agents including temperature and UV showed variable effects on the survival of FMD (O/pan Asia, A/Iran05, and SAT-2/2012) virus. Effect of temperature on (*in vitro)* replication of FMDV is represented in Table-1.

**Table-1:** Effect of heat and UV light on survival of foot and mouth disease virus serotypes “O,” “A”, and “SAT-2.”

Item for inactivation							

Heat				UV light			
	
Temperature exposure	time (min) for FMDV (O, A, and SAT −2)			Exposure time (min)	UV exposure		
					
					O	A	SAT-2
Temperature °C	15	30	45	15 min	+	+	+
				30 min	+	+	+
37°C	+	+	+	45 min	+	+	+
57°C	−	−	−	60 min	−	+	−
77°C	−	−	−	65 min	−	−	−
−ve control	+	+	+	−ve control	+	+	+
+ve control	−	−	−	+ve control	−	−	−

(-)=Means that the virus was complete inactivated, (+)=means that the virus was not inactivated, FMDV=Foot and mouth disease virus, UV=Ultraviolet, CPE=Cytopathic changes

The virus suspension when was exposed to heat treatment at 37°C was not inactivated after 15, 30, and 45 min of exposure, and showed CPE on BHK cell line. However when the virus suspension exposed to heat treatment at 57 and 77°C, it was got inactivating after 15, 30, and 45 min of the interaction time. These results showed that complete inactivation was done after exposure to temperature ≥57°C for 15 min.

Effects of UV treatment on survival of virus have been shown in Table-1. The virus exposed to UV was not inactivated after interaction time of 15, 30, and 45 min, and each of the virus samples showed CPE on the BHK-21 cell line. The inactivation of FMDV (O/pan Asia, A/Iran05, and SAT-2/2012) occurs within 60 min, but FMDV type (A) was inactivated within 65 min.

**Figure-1:**
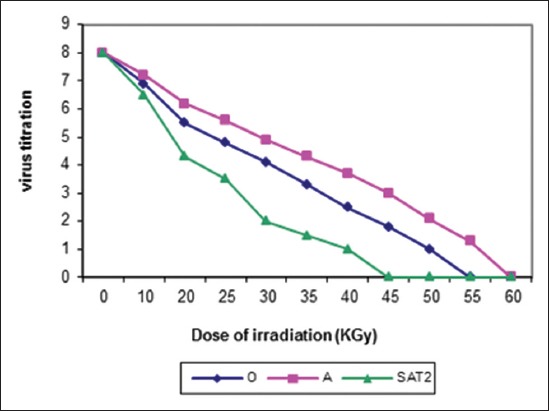
Effect of gamma irradiation on foot and mouth disease virus serotypes “O,” “A”, and “SAT-2.”

**Figure-2:**
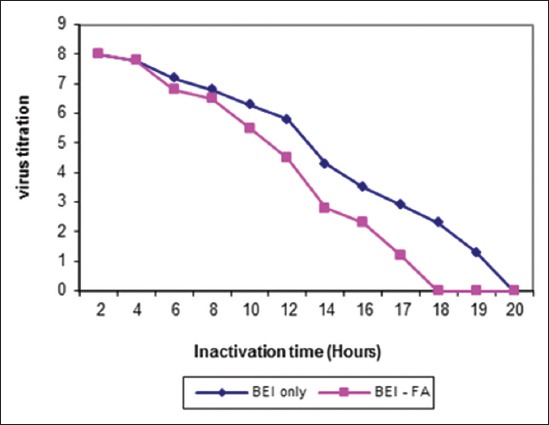
Inactivation kinetics of foot and mouth disease virus type O/pan Asia either binary ethyleneimine (BEI) only or BEI-FA at 37°C.

**Figure-3:**
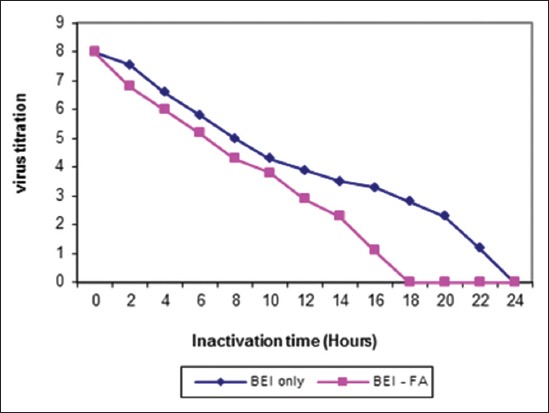
Inactivation kinetics of foot and mouth disease virus type A/Iran05 either binary ethyleneimine (BEI) only or BEI-FA at 37°C.

**Figure-4:**
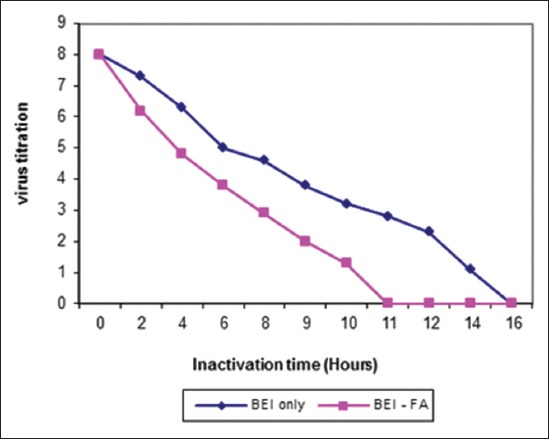
Inactivation kinetics of foot and mouth disease virus type SAT-2/2012 either binary ethyleneimine (BEI) only or BEI-FA at 37°C.

The ideal dose of gamma radiation for inactivation of FMDV (O/pan Asia, A/Iran 05, and SAT-2/2012) were (55, 60 and 45) kGy, respectively as shown in Figure-1. Inactivation of FMDV with binary obtained after (20, 24 and 16) hrs for (O-A and SAT-2), respectively while inactivation by binary with formaldehyde was (18, 19 and 11) hrs, respectively were shown in Figures-2-4 and Table-2.

**Table-2:** Scheme for FMDV strains inactivation time.

Inactivation factor	Time for inactivation FMDV strain		

	O	A	SAT-2
Heat	15 min at 57°C		
γ-radiation	Dose of irradiation 55 KGy	Dose of irradiation 60 KGy	Dose of irradiation 45 KGy
UV ray	252.7 nm for 60 min	252.7 nm for 65 min	252.7 nm 60 min
Binay	20 h at 37°C	24 h at 37°C	16 h at 37°C
Binay and formaldehyde	18 at 37°C	19 h at 37°C	11 h at 37°C

FMDV=Foot and mouth disease virus, UV=Ultraviolet

No CPE was observed by the virus negative control wells l as well as virus inactivated samples while the significant CPE was noted in positive control virus.

Table-3 demonstrated that the antigenicity of control virus before inactivation was 1/32, antigenicity was not changed after inactivation in cases gamma radiation and binary with formaldehyde and slightly decreased to (1/16) in case of binary only and became (1/2 and 1/4) by heat and UV inactivation, respectively.

**Table-3:** Complement fixation content of inactivated FMDV antigen strains before and after inactivation.

CF content of inactivated virus by									

Heat		γ-radiation		UV		BEI only		BEI with formaldehyde	
				
Before inactivation	After inactivation	Before inactivation	After inactivation	Before inactivation	After inactivation	Before inactivation	After inactivation	Before inactivation	After inactivation
1/32	1/2	1/32	1/32	1/32	1/4	1/32	1/16	1/32	1/32

FMDV=Foot and mouth disease virus, UV=Ultraviolet, BEI=Binary ethyleneimine

**Figure-5:**
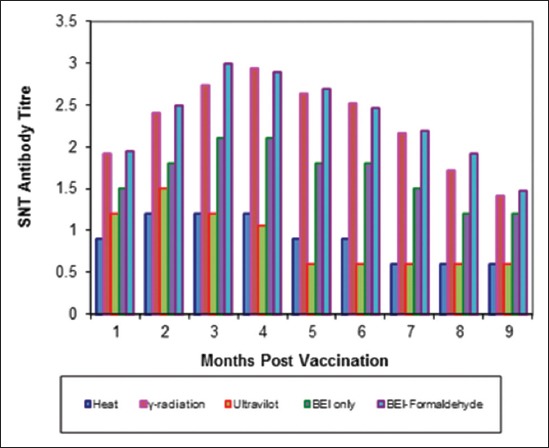
Mean foot and mouth disease (FMD) O/pan Asia antibody titers in calves vaccinated with different inactivated FMD vaccine using serum neutralization test.

**Figure-6:**
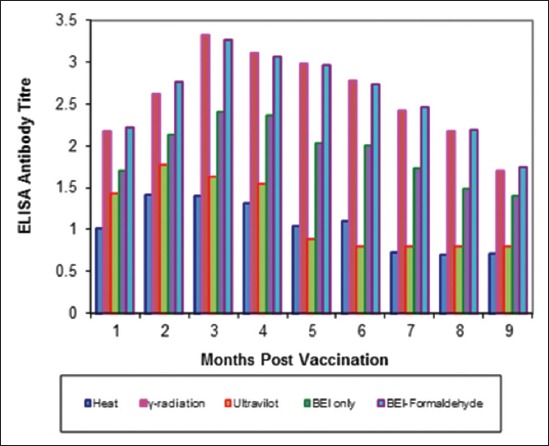
Mean foot and mouth disease (FMD) O/pan Asia antibody titers in calves vaccinated with different inactivated FMD vaccine using enzyme-linked immunosorbent assay.

**Figure-7:**
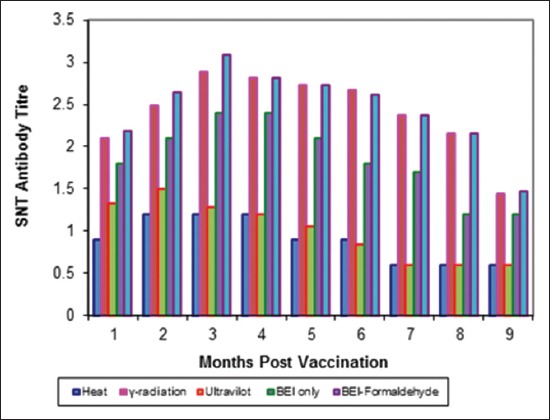
Mean foot and mouth disease (FMD) A/Iran05 antibody titers in calves vaccinated with different inactivated FMD vaccine using serum neutralization test.

**Figure-8:**
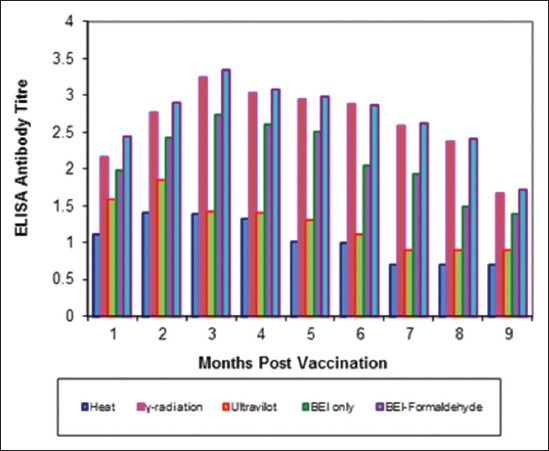
Mean foot and mouth disease (FMD) A/Iran05 antibody titers in calves vaccinated with different inactivated FMD vaccine using enzyme-linked immunosorbent assay.

**Figure-9:**
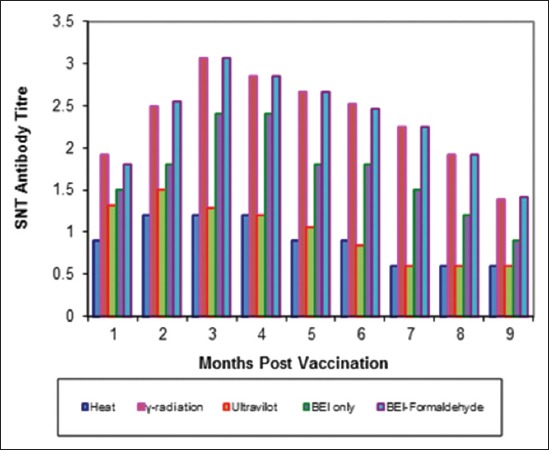
Mean foot and mouth disease (FMD) SAT-2 antibody titers in calves vaccinated with different inactivated FMD vaccine using serum neutralization test.

**Figure-10:**
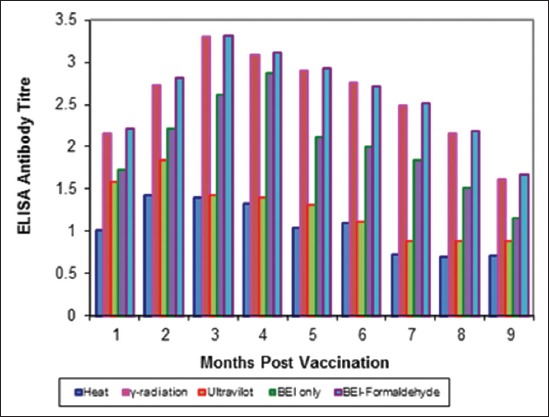
Mean foot and mouth disease (FMD) SAT-2 antibody titers in calves vaccinated with different inactivated FMD vaccine using enzyme-linked immunosorbent assay.

Tables-4-6 and Figures-5-10 demonstrated that the immune response of inactivated FMD vaccine by gamma radiation and binary with formaldehyde lasted to 9 months post-vaccination, while the binary (BEI) only still up to 8 months post-vaccination (MPV) but heat and UV inactivator vaccines were not effective.

**Table-4:** Mean FMD O/pan Asia antibody titers in calves vaccinated with different inactivated FMD vaccine using SNT and ELISA.

Inactivated FMDV vaccine by	FMD (SAT-2) antibody titer/month																	

	1 MPV		2 MPV		3 MPV		4 MPV		5 MPV		6 MPV		7 MPV		8 MPV		9 MPV	
								
	SNT	ELISA	SNT	ELISA	SNT	ELISA	SNT	ELISA	SNT	ELISA	SNT	ELISA	SNT	ELISA	SNT	ELISA	SNT	ELISA
Heat	0.9	1.01	1.2	1.42	1.2	1.4	1.2	1.32	0.9	1.04	0.9	1.1	0.6	0.72	0.6	0.7	0.6	0.71
γ-radiation	1.92	2.18	2.4	2.62	2.49	3.32	2.82	3.11	2.64	2.98	2.52	2.78	2.16	2.42	1.72	2.18	1.41	1.7
UV	1.2	1.43	1.5	1.78	1.2	1.63	1.05	1.54	0.6	0.89	0.6	0.8	0.6	0.8	0.6	0.8	0.6	0.8
BEI only	1.5	1.7	1.8	2.13	2.1	2.41	2.1	2.37	1.8	2.03	1.8	2.0	1.5	1.73	1.2	1.48	1.2	1.4
BEIformaldehyde	1.95	2.22	2.49	2.76	3.0	3.27	2.79	3.06	2.7	2.97	2.46	2.73	2.19	2.46	1.92	2.19	1.47	1.74

Antibody titers expressed in log_10_, protective serum neutralizing antibody titer 1.5, Protective ELISA antibody titer 1.9, FMDV=Foot and mouth disease virus, MPV=Month postvaccination, SNT=Serum neutralization test, ELISA=Enzymelinked immunosorbent assay, BEI=Binary ethyleneimine

**Table-5:** Mean FMD A/Iran 05 antibody titers in calves vaccinated with different inactivated FMD vaccine using SNT and ELISA.

Inactivated FMDV vaccine by	FMD (SAT-2) antibody titer/month																	

	1 MPV		2 MPV		3 MPV		4 MPV		5 MPV		6 MPV		7 MPV		8 MPV		9 MPV	
								
	SNT	ELISA	SNT	ELISA	SNT	ELISA	SNT	ELISA	SNT	ELISA	SNT	ELISA	SNT	ELISA	SNT	ELISA	SNT	ELISA
Heat	0.9	1.11	1.2	1.41	1.2	1.39	1.2	1.32	0.9	1.01	0.9	1.0	0.6	0.7	0.6	0.7	0.6	0.7
γ-radiation	2.1	2.16	2.49	2.77	2.03	3.24	2.82	3.03	2.73	2.95	2.67	2.88	2.37	2.59	2.16	2.38	1.44	1.66
UV	1.32	1.58	1.5	1.84	1.29	1.42	1.2	1.4	1.05	1.31	0.84	1.11	0.6	0.89	0.6	0.89	0.6	0.89
BEI only	1.8	1.98	2.1	2.42	2.4	2.73	2.4	2.61	2.1	2.51	1.8	2.04	1.7	1.93	1.2	1.48	1.2	1.39
BEIformaldehyde	2.19	2.44	2.64	2.89	3.09	3.34	2.82	3.07	2.73	2.98	2.61	2.86	2.37	2.62	2.16	2.41	1.47	1.72

FMDV=Foot and mouth disease virus, MPV=Month postvaccination, SNT=Serum neutralization test, ELISA=Enzymelinked immunosorbent assay, BEI=Binary ethyleneimine

**Table-6:** Mean FMD SAT-2 antibody titers in calves vaccinated with different inactivated FMD vaccine using SNT and ELISA.

Inactivated FMDV Vaccine by	FMD (SAT-2) antibody titer/month																	

	1 MPV		2 MPV		3 MPV		4 MPV		5 MPV		6 MPV		7 MPV		8 MPV		9 MPV	
								
	SNT	ELISA	SNT	ELISA	SNT	ELISA	SNT	ELISA	SNT	ELISA	SNT	ELISA	SNT	ELISA	SNT	ELISA	SNT	ELISA
Heat	0.9	1.01	1.2	1.42	1.2	1.4	1.2	1.32	0.9	1.04	0.9	1.1	0.6	0.72	0.6	0.7	0.6	0.71
γ-radiation	1.92	2.16	2.49	2.73	3.06	3.3	2.85	3.09	2.67	2.9	2.52	2.76	2.25	2.49	1.92	2.16	1.38	1.62
UV	1.32	1.58	1.5	1.84	1.29	1.42	1.2	1.4	1.05	1.31	0.84	1.11	0.6	0.89	0.6	0.89	0.6	0.89
BEI only	1.5	1.73	1.8	2.22	2.4	2.61	2.4	2.87	1.8	2.12	1.8	2.0	1.5	1.84	1.2	1.51	0.9	1.15
BEIformaldehyde	1.8	2.21	2.55	2.81	3.06	3.32	2.85	3.11	2.67	2.93	2.46	2.72	2.25	2.51	1.92	2.18	1.41	1.67

FMDV=Foot and mouth disease virus, MPV=Month postvaccination, SNT=Serum neutralization test, ELISA=Enzymelinked immunosorbent assay, BEI=Binary ethyleneimine

## Discussion

The inactivation methods of FMDV include the use of formaldehyde, ethyleneimine, and propylene imine. Both of these agents have some residues in the final products, some are toxic, and some cause allergic responses in animals [28].

This research studied the inactivation of FMD virus strains using different methods. Temperature treatment of FMDV at 37°C for 15, 30, and 45 min had an undetectable effect on FMDV serotype (O/pan Asia, A/Iran05, and SAT-2/2012). These results came parallel to that mentioned by Razdan *et al*. [37] who recorded that infectivity titers of FMD “O” virus in maintenance cell culture medium was reduced by 2 log units on storage at 37°C for 12 h. It indicates that virus temperature interaction time at 37°C has an inverse effect on the virus infectivity. Heat treatments at 57°C and 77°C for a period of 15 min or longer time are suitable for complete inactivation of the virus. Heat treatment at 60°C inactivated FMD virus [38,39]. Heating the virus suspension at 56°C for 60 min reduces its infectivity [40]. At high temperature, may be done the destruction of virus receptors, which ultimately declined its infectivity. The “O,” “C,” and “Asia-1” serotypes of FMD virus may inactivated at 54°C for 1 h [41].

The UV light had no effect on the virus after interaction time of 15, 30, and 45 min and the virus produced CPE on BHK cell line. The inactivation of FMDV (O/pan Asia and SAT-2/2012) occurs within 60 min and 65 min for type (A). Our findings are in aligned with Nuanualsuwan *et al*. [21] and Rabia *et al*. [42] who observed that vesicular stomatitis and FMDV were inactivated when exposed to UV light source. These findings are not in aligned with Hazem [43] who mentioned that the virus had resistant properties to UV light.

The ideal dose for inactivation of FMDV with gamma ray was obtained between 45 and 60 kGy (according to virus strain). Complete inactivation was determined with 45 kGy for FMDV (SAT-2), 55 kGy for FMDV (O), and 60 kGy for FMDV (A). These results were an agreement with Sedehl *et al*. [44] who recorded that the ideal dose range of gamma ray for FMD virus inactivation with virus titration 10^7.5^ TCID_50_/ml was obtained between 40 and 44 kGy. Furthermore, the results of safety test for irradiated samples with gamma ray doses were suitable because there is no detection to CPE after three times in cell culture.

Inactivated virus with BEI showed complete inactivation of FMDV (O/pan Asia, A/Iran05, and SAT-2/2012) within 20, 24 and 16 hrs, respectively, while inactivated virus using combination of BEI with formaldehyde at the same conditions showed complete inactivation of FMDV (O/pan Asia, A/Iran05, and SAT-2/2012) within 18, 19, and 11 hrs, respectively.

These results agree with Ali *et al*. [45] who mentioned that inactivation only with BEI in concentration of 0.1 M, FMD type (A) virus was completely inactivated after 15 h, and FMDV type O_1_ was completely inactivated after 14 h. Furthermore, agree with Bahneman [26]; Aarthi *et al*. [46]; Ismail *et al*. [47] whose showed that the complete inactivation of FMDV type SAT-2 with no residual virus was detected.

The antigenicity of control virus before and after inactivation was 1/32 in inactivation viruses with gamma radiation or binary with formaldehyde and reduced to (1/16) using BEI alone and declined to (1/2-1/4) for heat and UV inactivation, respectively. These results agreed with those of [45,48] who mentioned that the CF content for the inactivated virus (Type O) with BEI only was reduced from 1/32 to 1/16 after inactivation, also where was no change of antigenicity with combination of BEI-formaldehyde with the (O and A) virus types. Our results agree with Bahneman [25] who mentioned that the complement fixation of 2 types of virus (O and A) using BEI-formaldehyde as inactivator were not changed before and after inactivation process and come in agreement with the results obtained by Ali *et al*. [45].

All inactivated viruses showed, no residual viable virus as detected in tissue cultures or in baby mice. Therefore, the inactivated virus could be with antigenicity and good safety test results in the preparation of inactivated vaccine. Now the formulated vaccine is inoculated to animals and is studied the animals immunization by seronutralization and ELISA method.

The immune response of inactivated FMD vaccines by gamma radiation and binary with formaldehyde lasted to 9 months post-vaccination while the binary (BEI) only still up to 8 MPV but heat and UV-inactivated vaccines were not effective these results were agreement with Ljungman *et al*. [49] who recorded that the improved safety vaccines were suitable for the rapidly increasing immunocompromised.

Finally, we concluded that the gamma radiation could be considered a good new inactivator, inducing the same immune response of inactivated vaccine with binary with formaldehyde.

## Authors’ Contributions

SEM inoculation of cell culture with FMDV serotype SAT-2/2012 and sharing in titeration and inactivation process by different methods for SAT-2/2012, AIH inoculation of cell culture with FMDV serotype O/pan Asia and sharing in titration and inactivation process by different methods for O/pan Asia, WMGE inoculation of cell culture with FMDV serotype A /Iran05 and sharing in titeration and inactivation process by different methods for A /Iran05, EEI preparation of BHK13 clone 21 for virus inoculation, titration and SNT also sharing in inactivation process by different methods, write the manuscript and follow-up the steps for publication and HMF sharing in inactivation process by different methods, confirm the complete inactivation process by different methods, measure the antigen content and apply SNT, ELISA and write the manuscript.
